# End-of-surgery MRI following pediatric neuro-oncological tumor resection

**DOI:** 10.1007/s00381-026-07267-z

**Published:** 2026-05-26

**Authors:** Timothy Mueller, Eike Immo Piechowiak, Jonathan Wermelinger, Sarah Ricchizzi, Andreas Raabe, Katharina Lutz, Philippe Schucht

**Affiliations:** 1 Department of Neurosurgery, Inselspital, Bern University Hospital, University of Bern, Bern, Switzerland; 2 Institute of Diagnostic and Interventional Neuroradiology, Inselspital, Bern University Hospital, University of Bern, Bern, Switzerland

**Keywords:** End-of-surgery MRI, Intraoperative MRI, Brain tumor, Pediatric neurosurgery, Extent of resection

## Abstract

**Purpose:**

Magnetic resonance imaging (MRI) is routinely used to assess extent of resection after pediatric brain and spine tumor surgery and is commonly performed intraoperatively (ioMRI) or postoperatively (postopMRI). This study describes the implementation, feasibility, and workflow characteristics of end-of-surgery MRI (eosMRI), performed after wound closure but before termination of general anesthesia within a single-center pediatric neurosurgical practice and reports procedural and patient-related parameters observed over a 10-year period.

**Methods:**

We conducted a retrospective, single-center observational study including all pediatric brain and spine tumor surgeries performed with eosMRI, ioMRI, or postopMRI between July 2013 and September 2022. MRI modality selection followed institutional workflow patterns and surgeon judgment rather than predefined allocation criteria. Recorded variables included anesthesia time, surgery duration, imaging duration, patient age, tumor location, imaging-guided additional resection, and extent of resection (EOR).

**Results:**

A total of 129 surgeries in 106 children (mean age 9 years, range 0.3–18) were analyzed: 45 eosMRI (35%), 16 ioMRI (12%), and 68 postopMRI (53%). Within this workflow, eosMRI and ioMRI were associated with longer anesthesia times compared to postopMRI, whereas total surgery time was prolonged only in the ioMRI group. Imaging duration did not differ significantly across modalities. Patients managed with eosMRI were younger and more frequently had infratentorial tumors, reflecting institutional selection patterns. Extent of resection (EOR) distribution did not differ statistically between modalities.

**Conclusion:**

This retrospective single-center experience demonstrates the feasibility of eosMRI within a pediatric neurosurgical workflow, particularly regarding operative logistics and anesthesia exposure. These descriptive findings may inform perioperative imaging considerations in comparable institutional settings for pediatric neuro-oncological surgery.

## Introduction

Brain and spinal tumors are the most common solid tumors in children and a leading cause of cancer-related mortality. Surgical resection is the mainstay of treatment, and the extent of resection (EOR) strongly influences overall survival (OS) [1]. Postoperative magnetic resonance imaging (postopMRI), performed within 72 h, is considered the gold standard for assessing residual tumor burden, guiding decisions for reoperation, and planning adjuvant chemo- and radiotherapy [1, 2].

However, postopMRI remains challenging in the pediatric population, as maintaining immobility during lengthy and noisy scans is difficult for young children. Sedation or general anesthesia is often required, adding risk and complexity. Typically, anesthesia is necessary for children under 6–7 years, while most older children (> 7 years) can cooperate adequately during scanning [3]. Additionally, if substantial residual tumor is detected, a second surgery may become necessary, exposing the patient to additional surgical and cumulative anesthetic risks [4]. These challenges highlight the need for alternative imaging approaches that enhance patient comfort, provide immediate feedback, and reduce repeat procedures.

In recent years, intraoperative MRI (ioMRI) has gained recognition for its potential benefits in pediatric brain tumor surgery [5–8]. By facilitating immediate assessment of EOR, ioMRI allows for timely interventions if tumor remnants are detected, ultimately enhancing surgical outcomes. However, several limitations have been widely acknowledged, including the time-consuming nature of the procedure, workflow disruption, and the complex patient positioning required for infratentorial tumors [7, 9, 10]. In addition, ioMRI is associated with substantial costs and limited resource efficiency: even in a two-room set-up, a single ioMRI examination may reduce scanner capacity by approximately four standard outpatient or inpatient MRI slots on average (internal institutional data). From a global perspective, the availability and feasibility of implementation remains a significant obstacle to widespread adoption [9, 10].

End-of-surgery MRI (eosMRI), defined as MRI performed under the same general anesthesia without a skull clamp in the supine position immediately after definitive surgical closure, has been already described in individual cases within ioMRI studies and can be used as a potential alternative to ioMRI [7].

EosMRI was initially introduced at our department due to the lack of ioMRI and to avoid a second anesthesia in young children requiring postoperative imaging, thereby improving patient comfort. It has remained in routine use even after the introduction of ioMRI in 2017, providing an approach to streamline the surgical workflow.

This study describes the use of eosMRI within our institutional workflow over a 10-year period and reports procedural characteristics across cases in which eosMRI, ioMRI, or postopMRI were applied.

## Methods

### Patient selection

We retrospectively screened all patients aged ≤ 18 who underwent brain or intradural spinal tumor surgery at the Department of Neurosurgery at the Inselspital (University Hospital of Bern) between 01/2013 and 12/2022.

### Data acquisition

Demographic data, tumor location and histology, and imaging modality (eosMRI, ioMRI, or postopMRI) were extracted from medical records. Surgery, anesthesia, and imaging times were obtained from the operating room time-tracking system. Pre- and postoperative MRI were compared to determine the EOR.

### Imaging modalities: postopMRI, eosMRI and ioMRI

*PostopMRI* was performed within 72 h after tumor resection, following standard clinical protocols for early postoperative imaging. Scans were acquired on either a 1.5 T or 3 T MRI system (Avanto, Avanto Fit, Aera, Skyra Fit, Verio or Vida; Siemens Healthineers, Erlangen, Germany), depending on scanner availability. Imaging was performed under general anesthesia for younger or uncooperative patients, whereas older children underwent the procedure awake whenever feasible.

*EosMRI* was performed immediately after completion of the surgical procedure and definitive skin closure. The patient remained under general anesthesia, was repositioned supine, and transferred to a standard MRI suite for non-sterile imaging. Scans were acquired on either a 1.5 T or 3 T MRI system (Avanto, Avanto Fit, Aera, Skyra Fit, or Vida; Siemens Healthineers, Erlangen, Germany). After eosMRI, patients were transferred to the pediatric intensive care unit or returned to the operating theatre if clinically indicated.

*IoMRI* was performed in a dedicated two-room ioMRI suite. This unit consisted of a fully equipped operating theatre directly connected to a 1.5 T MRI scanner room (Aera; Siemens Healthineers, Erlangen, Germany) via a sliding door, allowing patient transfer while maintaining a sterile environment. After temporary skin closure, the patient remained under general anesthesia and was transferred in a sterile fashion and still fixed in the skull clamp to the MRI scanner. Following ioMRI, the patient was either transferred to the pediatric intensive care unit for recovery or, if residual tumor was detected, returned to the operating theatre for further resection before final wound closure.

### Imaging modality selection

At our department, the surgical team could choose among several MRI-based modalities for resection control. Between January 2013 to December 2016, the available options included postopMRI and eosMRI. In January 2017, ioMRI was introduced as an additional modality.

Modality selection was based on surgeon judgment rather than predefined criteria. Although no standardized allocation protocol was applied, modality selection in most cases followed recurring clinical considerations, including the anticipated need for further tumor resection, patient age, tumor location and positioning constraints, and ioMRI availability.

**Fig. 1:**
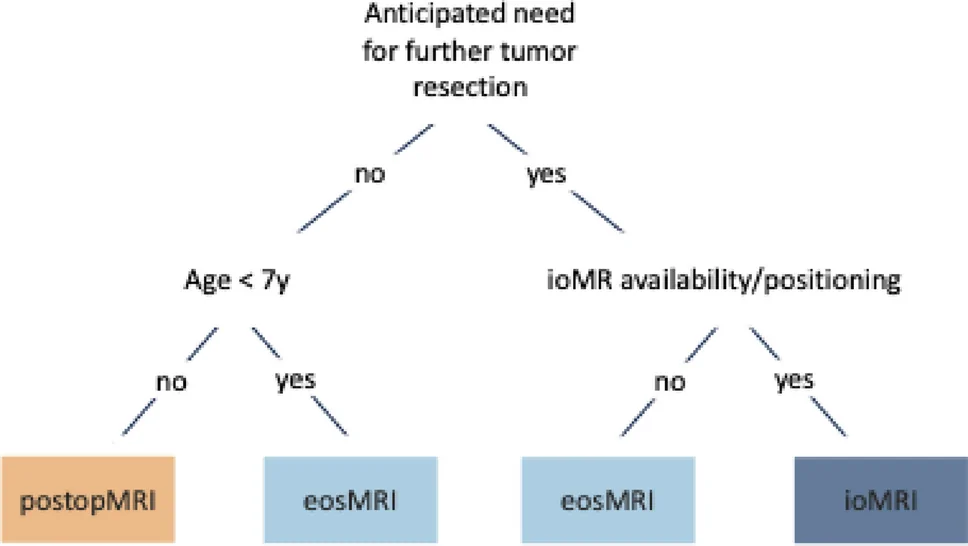
Schematic illustration of the institutional decision-making framework that commonly guided MRI modality selection during the study period. The diagram summarizes routine clinical practice rather than a prospectively defined or validated allocation algorithm

A schematic overview of the institutional decision-making framework commonly applied during the study period is provided in Fig. 1.

### Definitions of imaging and procedure times

*Total surgery time* was defined as the interval from skin incision to definitive skin closure, including the duration of MRI acquisition in cases with ioMRI. Any return to surgery after eosMRI was included in the total surgery time.

*Total anesthesia time* was defined as the interval from induction of general anesthesia (GA) to its discontinuation at the end of surgery. Anesthesia time related to postopMRI imaging was recorded separately.

For eosMRI and ioMRI, *imaging time* was measured from skin closure to completion of the final MRI sequence, including transfer and preparation time. For postopMRI, the *imaging time* under anesthesia was defined as the interval from induction of anesthesia to completion of the final MRI sequence, in patients requiring GA.

### Statistical analysis

Descriptive statistics were computed for continuous and categorical variables. The normality of continuous variables was assessed using the Shapiro–Wilk test. As normality was rejected, non-parametric tests were applied.

Exploratory comparisons of continuous variables across MRI types were analyzed using the Kruskal–Wallis test. If significant, post-hoc pairwise comparisons were performed using the Wilcoxon rank-sum test. Holm’s method was used to adjust p-values for multiple testing. For categorical variables, exploratory group comparisons were assessed using Fisher’s Exact Test, since counts in contingency table cells were below five. Pairwise Fisher’s exact tests with Holm’s correction were used where appropriate.

Given the observational study design and non-randomized modality allocation, all statistical analyses are descriptive and do not imply causal inference.

Statistical significance was set at p < 0.05, and different levels of significance were marked with asterisks (*p < 0.05, **p < 0.01, ***p < 0.001). All statistical analyses were conducted using R Studio (Version 4.3.3, R Studio Inc.)

## Results

### Patient characteristics

**Fig. 2:**
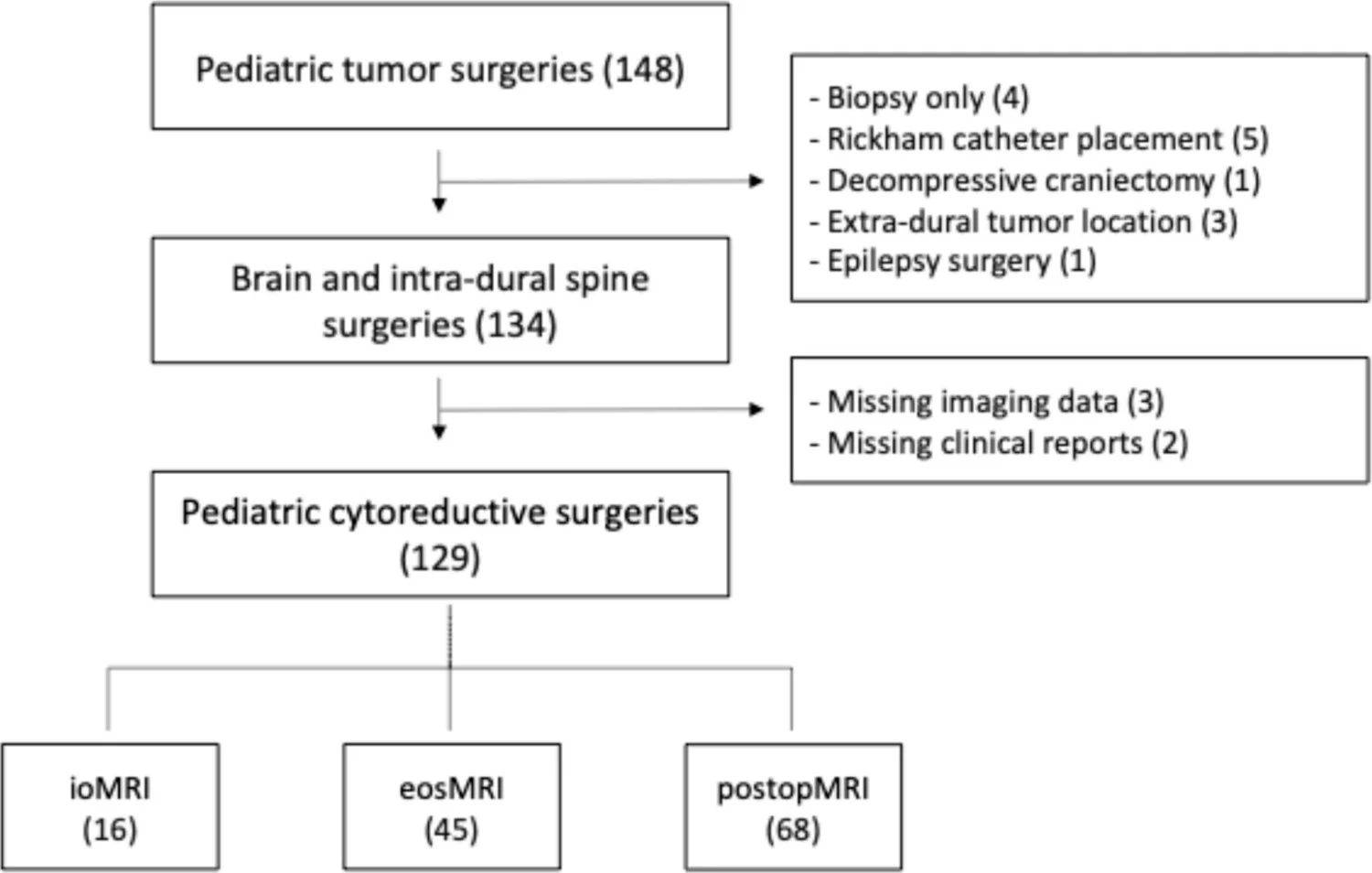
Flowchart for study inclusion and exclusion criteria of 148 pediatric tumor surgeries screened, 129 procedures met eligibility criteria and were included in the final analysis. *eosMRI:* end-of-surgery MRI, *ioMRI:* intraoperative MRI, *MRI:* magnetic resonance imaging, *postopMRI:* postoperative MRI

**Fig. 3:**
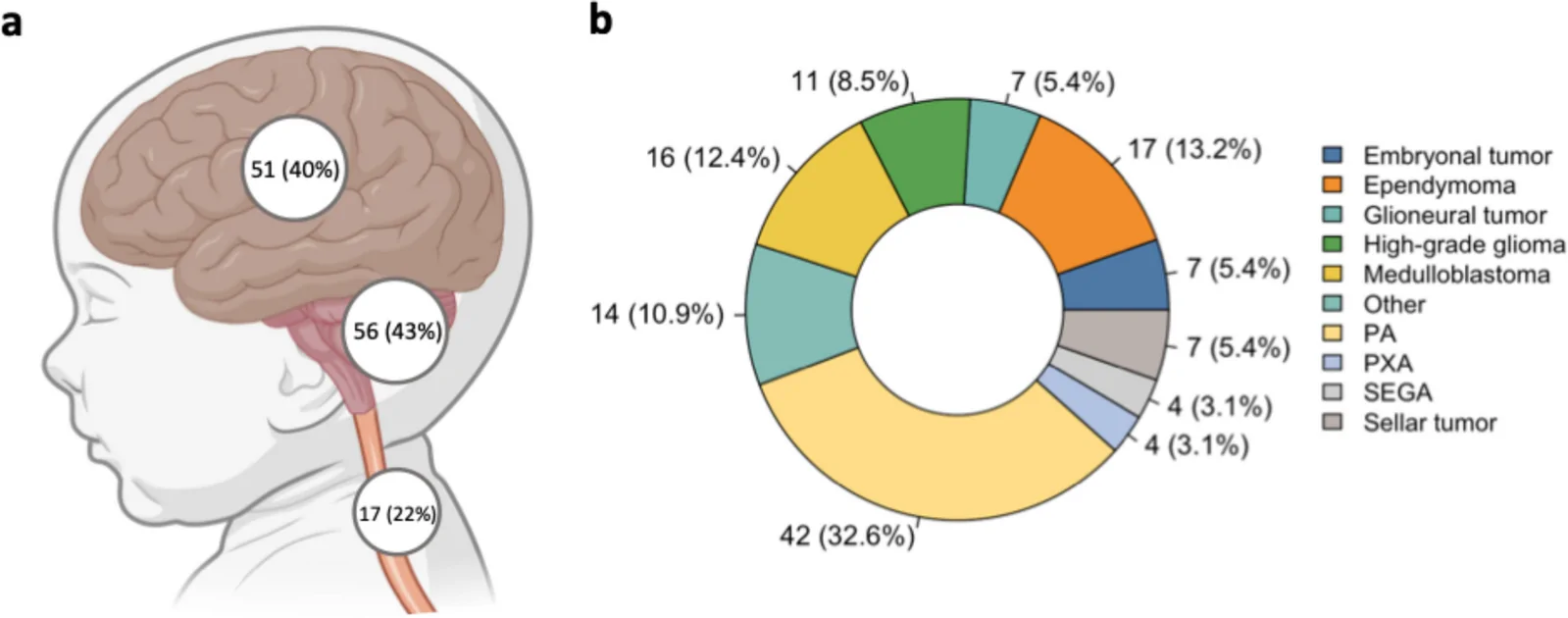
Distribution of tumor locations and histopathological entities. **a** Tumor location stratified by supratentorial (brown), infratentorial (red), and spinal (orange) regions (created with BioRender.com). **b** Distribution of tumor types within the study cohort (*n* = 129). *PA*: pilocytic astrocytoma, *PXA*: pleomorphic xanthoastrocytoma, *SEGA*: subependymal giant cell astrocytoma

Between 01/2013 and 12/2022, 116 pediatric patients underwent 148 brain or spine tumor surgeries, of which 106 patients and 129 procedures were included due to complete documentation (Fig. 2). The cohort comprised 83 female (63.4%) and 48 male (36.6%) patients, with a mean age of 9.2 years (range 0.3–17.9). Tumor entities and locations are shown in Fig. 3.

### Imaging modality

**Fig. 4:**
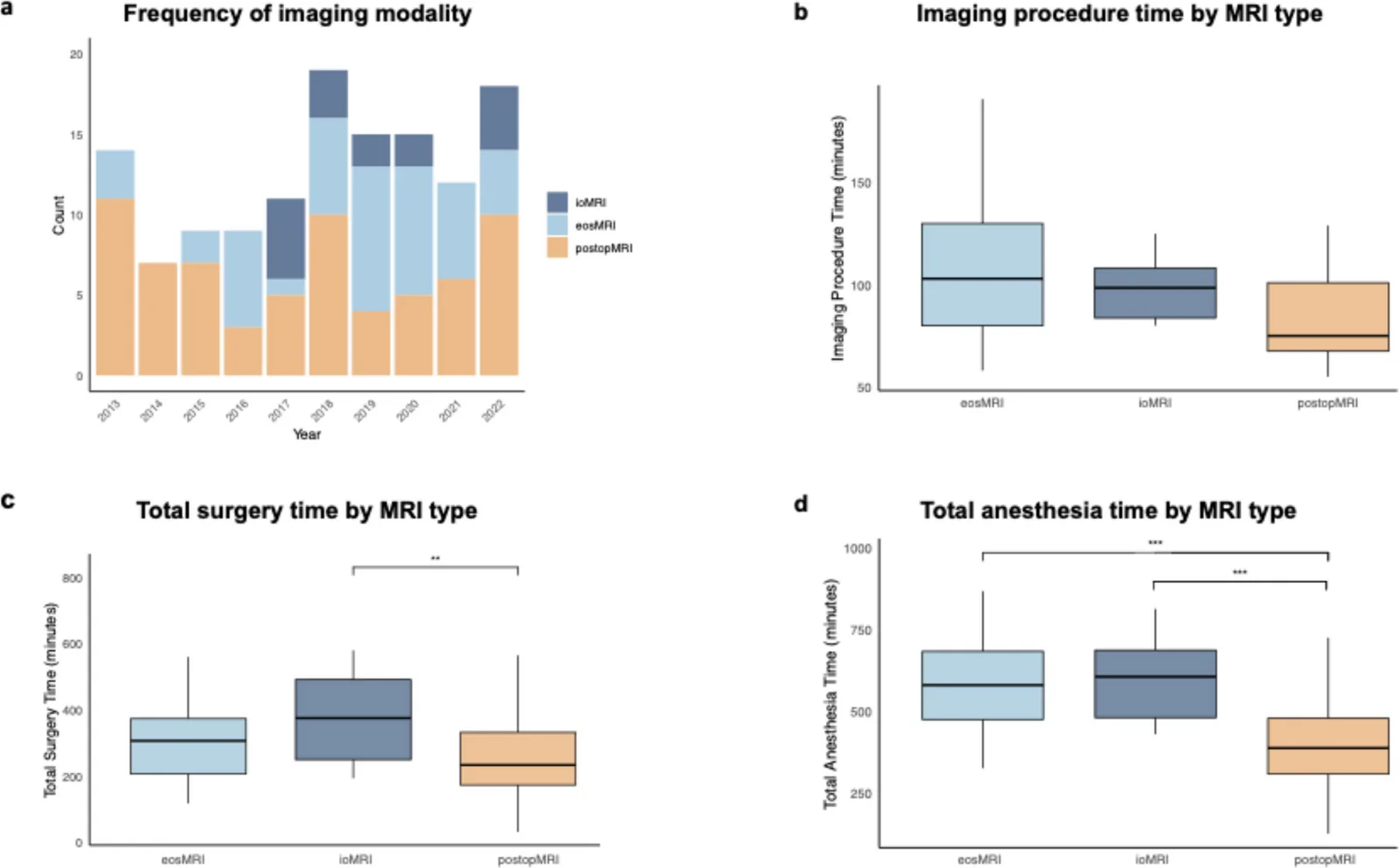
Utilization of MRI modalities and procedural time metrics. **a** Annual distribution of imaging modalities between July 2013 and September 2022. **b** Imaging time by MRI type, measured from the start to the end of MRI acquisition under general anesthesia. **c** Total surgery time by MRI type, defined as the interval between skin incision and definitive skin closure (including MRI acquisition time for ioMRI cases). **d** Total anesthesia time by MRI type, measured from induction to discontinuation of general anesthesia. ** statistically significant at *p* < 0.01 and *** statistically significant at *p* < 0.001 using Kruskal–Wallis and Wilcoxon post-hoc test

The utilization of eosMRI, ioMRI, and postopMRI between 2013 and 2022 is illustrated in Fig. 4a. Although ioMRI was introduced in January 2017, eosMRI remained in routine clinical use.

### Total imaging, surgery and anesthesia time

Eleven patients underwent postopMRI under general anesthesia (median 92 min, range 67–185). Imaging time did not differ significantly between MRI groups (Kruskal–Wallis test, *p* = 0.08) (Fig. 4b). A significant difference in total surgery time was observed between MRI groups (Kruskal–Wallis test, *p* < 0.001) (Fig. 4c). Post-hoc analysis revealed that surgeries in the ioMRI group were significantly longer than those in the postopMRI group (*p* < 0.01), whereas the difference between eosMRI and postopMRI did not reach statistical significance (*p* = 0.14).

A significant difference in total anesthesia time for surgery was observed among the three MRI groups (Kruskal–Wallis test, p < 0.001) (Fig. 4d). Post-hoc analyses showed significantly longer anesthesia times for eosMRI and ioMRI compared with postopMRI (both *p* < 0.001) (Fig. 4d). When total anesthesia time for surgery was combined with anesthesia time for postoperative MRI, no significant difference was observed between the postopMRI group and the ioMRI or eosMRI groups (Kruskal–Wallis test, *p* = 0.15).

### Tumor location and age distribution

**Fig. 5:**
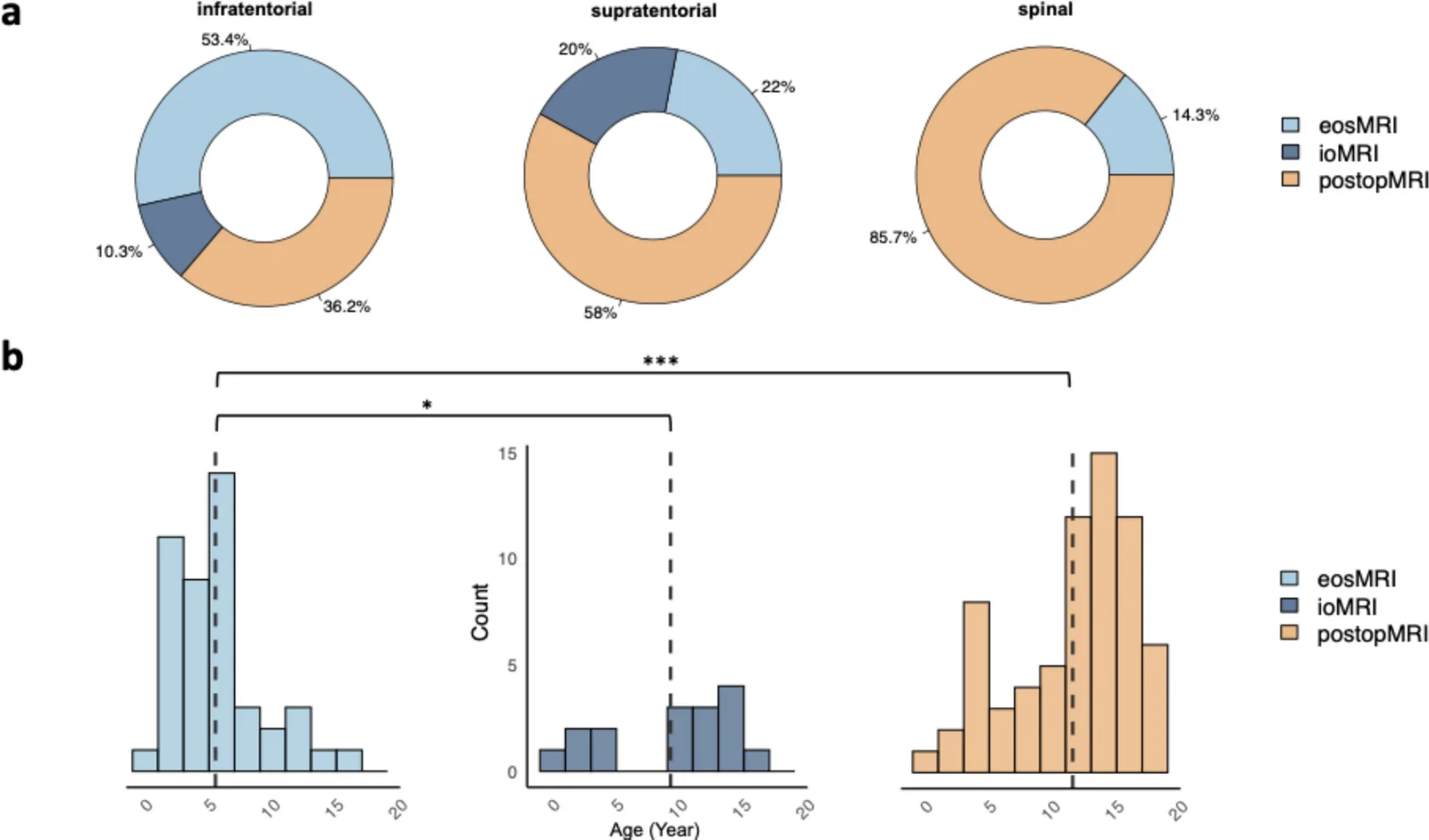
Distribution of MRI modality according to tumor location and patient age. **a** Distribution of imaging modality by tumor location (infratentorial, supratentorial, and spinal). **b** Age distribution for patients undergoing eosMRI, ioMRI, and postopMRI, with median values indicated by dashed lines. * statistically significant at *p* < 0.05 and *** statistically significant at *p* < 0.001 using Kruskal–Wallis and Wilcoxon post-hoc test

The distribution of tumor location across MRI modalities showed statistically detectable differences within the cohort (Fisher’s exact test, *p* < 0.001) (Fig. 5a). Infratentorial tumors were more frequently managed with eosMRI, whereas supratentorial and spinal tumors were more commonly managed with postopMRI (Fig. 5a).

Age distribution also varied across modalities (Kruskal–Wallis test, p < 0.001) (Fig. 5b). The median age was 5.2 years (0.6–15.4) in the eosMRI group, 11.1 years (0.8–15.2) in the ioMRI group, and 12.3 years (0.2–18.0) in the postopMRI group.

These differences reflect institutional modality selection patterns during the study period rather than modality-specific effects.

### Extent of tumor resection

The distribution of EOR was not significantly different across the MRI groups (Fisher’s exact test, *p* = 0.13) (Table 1). Further tumor resection (during same surgery) was performed in 5 out of 16 (31%) children following ioMRI (Table 1). In the eosMRI group, 1 out of 45 (6%) patient was returned immediately to the operating room for further tumor resection, while another underwent reoperation 11 days later following the final histopathological analysis. Within the postopMRI group, 4 out of 68 patients (6%) were reoperated, at a median of 3.8 days (range: 1–7 days) after surgery (Table 1).

**Table 1:** Surgical characteristics and resection patterns according to MRI modality

Surgical outcome		ioMRI (*n* = 16)	eosMRI (*n* = 45)	postopMRI (*n* = 68)	*p*-value	Overall (*n* = 129)
Further tumor resection (during same surgery)		5	1	-	< 0.001^a^	6
Reoperation (second surgery)		0	1	4	0.28^a^	5
Time to reoperation (median [range], days)		0	11	5 (1–7)	0.02^b^	2.4 (1–11)
EOR	GTR	11	34	36	0.13^a^	81
	NTR	5	10	26		41
	STR	0	1	6		7

^a^Fisher’s Exact test, ^b^the Kruskal–Wallis test followed by pairwise post-hoc tests where appropriate. *EOR* Extent of resection, *GTR* Gross total resection, *NTR* Near total resection, S*TR* Subtotal resection

## Discussion

Although intraoperative and postoperative MRI have been extensively discussed in the literature, the structured application of eosMRI in pediatric neuro-oncological surgery has received comparatively little attention.

In this retrospective single-center analysis, we therefore focused on describing the implementation, feasibility, and practical role of eosMRI within our institutional perioperative imaging workflow.

### Imaging guidance in surgical decision-making

In our cohort, additional tumor resection during the same procedure was performed in 31% of ioMRI cases compared with 6% of eosMRI cases. Reported rates of additional resection following ioMRI in the literature range between 20 and 40% in pediatric tumor surgery [5, 11], which is consistent with our observations. Given the non-standardized modality allocation, the difference between the rather low additional resection rate observed for eosMRI most likely reflects the selective use of ioMRI in cases with a higher anticipated likelihood of residual tumor rather than modality-specific performance. IoMRI was typically employed in situations where continued resection was considered probable, whereas eosMRI was more often used when immediate resection control was desired without an expectation of further surgical extension.

Beyond workflow considerations, surgical decision-making in pediatric neuro-oncology is increasingly tumor entity-specific. For example, the prognostic relevance of residual disease differs between tumor types such as medulloblastoma [12] and ependymoma [13]. In such contexts, the anticipated need for intraoperative extension of resection may vary, and imaging strategies may therefore require individualized consideration. These tumor-specific outcome implications, however, were not addressed in the present analysis.

### Balancing total surgery, anesthesia and imaging time

IoMRI is known to prolong total anesthesia time [5, 8, 14], without increasing surgical complication rates [5, 8, 11]. In our cohort, eosMRI prolonged anesthesia time to a similar extent as ioMRI. Although both ioMRI and eosMRI were associated with longer anesthesia times, total surgery time was significantly increased only in the ioMRI group compared with postopMRI. This finding is attributable to the prolonged interval until definitive skin closure and the higher rate of continued surgery following intraoperative imaging, which extends operative duration. In contrast, eosMRI was not associated with a significant increase in total surgery time, potentially reducing risks related to prolonged immobilization in the Mayfield skull clamp, such as pressure injuries reported previously [15].

### Imaging considerations in young children and anesthesia exposure

General anesthesia is routinely required for MRI in children under seven years, whereas older children can usually undergo imaging awake [3]. Repeated anesthesia exposure remains a concern due to potential neurocognitive effects during early development [4]. In our cohort, patients undergoing eosMRI were significantly younger than those managed with ioMRI or postopMRI. This age distribution reflects institutional selection practices, in which eosMRI was preferentially used to avoid an additional postoperative anesthesia. However, the present study, was not designed to assess age-specific outcome effects.

### Addressing patient positioning challenges in infratentorial tumor surgery

Complex patient positioning, especially in infratentorial tumor surgery combined with ioMRI, presents unique challenges that necessitate careful preoperative planning [14]. These challenges arise from the fixed design of surgical tables, the limited aperture size of ioMRI scanners, and the constraints of head fixation devices, which often restrict optimal neck flexion and head orientation [11, 14–16]. Such limitations are less relevant with eosMRI and may therefore influence modality selection. This pattern is reflected in our cohort, where infratentorial tumors were more frequently managed with eosMRI due to positioning challenges rather than with ioMRI. This distribution reflects institutional workflow considerations in situations where positioning constraints reduced the practical feasibility of ioMRI.

### Optimizing neuroradiological resources

At our tertiary neuroradiology center, equipped with six operational MRI scanners, eosMRI examinations can typically be scheduled with minimal impact on routine workflow. In contrast, conducting a single ioMRI examination within our two-room setup substantially reduces scanner availability, effectively replacing four standard outpatient or inpatient MRI studies on average. This reduction is largely attributable to extended preparation times and increased MRI safety requirements in the intraoperative setting. From this point of view, eosMRI represents an efficient approach to optimizing MRI resource utilization in high-volume centers. However, this advantage is highly dependent on institutional infrastructure. In centers with multiple MRI scanners and a two-room ioMRI setup, eosMRI can be flexibly integrated into the perioperative workflow and maintain a balance between surgical imaging demands and routine diagnostic throughput.

## Limitations

Several limitations of this study warrant consideration.

First, the observational and retrospective design, combined with non-randomized modality allocation, substantially limits causal inference and precludes direct comparison between MRI modalities. Imaging selection was influenced by institutional workflow patterns and clinical judgment rather than predefined criteria, resulting in confounding by indication. Consequently, differences in procedural characteristics, resection patterns, or reoperation rates reflect workflow-driven findings rather than modality-specific effects. In addition, ioMRI was introduced during the study period, leading to temporal heterogeneity in modality availability that may have further influenced selection patterns and case distribution.

The relatively small subgroup sizes limit statistical power. Finally, all imaging and surgical decisions were made within a single tertiary referral center, which may restrict external generalizability.

The decision tree presented in this study reflects institutional practice during the study period and should be interpreted as a descriptive summary of clinical workflow rather than as a data-derived or validated allocation algorithm.

## Conclusion

This retrospective single-center study describes the integration of eosMRI into a pediatric neuro-oncological surgical workflow over a 10-year period. Within our institutional setting, eosMRI was feasible and allowed immediate postoperative imaging under the same anesthesia without prolonging surgical time. This approach may be of particular practical relevance in pediatric neuro-oncological surgery where minimizing repeated anesthesia exposure and enhancing patient comfort are important considerations.

## Data Availability

The data supporting the findings of this study are available from the corresponding author upon reasonable request.

## Abbreviations

MRI: Magnetic resonance imaging
ioMRI: Intraoperative MRI
postopMRI: Postoperative MRI
eosMRI: End-of-Surgery MRI
EOR: Extent of Resection
GTR: Gross total resection (> 98%)
NTR (> 90%): Near-total resection (> 90%)
STR (< 90%): Subtotal resection (< 90%)
OS: Overall survival
